# Neighborhood Polluting Infrastructure and Cognitive Health in Racially Diverse Older Adults

**DOI:** 10.1093/geroni/igaf122.3977

**Published:** 2025-12-31

**Authors:** Vivian Ku, Ketlyne Sol, Monica Walters, Noah Green, Laura Zahodne

**Affiliations:** Boston University, Boston, Massachusetts, United States; University of Miami, Boca Raton, Florida, United States; University of Michigan, Ann Arbor, Michigan, United States; University of Michigan, Ann Arbor, Michigan, United States; University of Michigan, Ann Arbor, Michigan, United States

## Abstract

Pollution exposure relates to worse cognitive health. Modifiable sources of pollution may serve as an intervention target, but less is known about how polluting infrastructure in the built environment relate to distinct cognitive domains and racial disparities. 772 adults ages 55+ from the Michigan Cognitive Aging Project completed a neuropsychological battery measuring five cognitive domains (episodic memory, executive function, processing speed, language, visuospatial function). Participants’ addresses were geocoded and linked to data from the National Neighborhood Data Archive. Polluting infrastructure was operationalized at the census-tract level as the presence of Toxic Release Inventory (TRI) sites and total highway length. Generalized estimating equations accounting for geographic clustering modeled relationships between polluting infrastructure and cognition, controlling for individual-level and neighborhood-level covariates. The presence of TRI sites related to worse language (β=-.140, p=.031), while greater total highway length related to worse executive function (β=-.007, p=.036) and language (β=-.011, p=.016). Race modified the relationship between total highway length and visuospatial function (β=-.024, p=.008). Greater total highway length related to better visuospatial function only among non-Hispanic Black participants (β=.027, p=.038). Highways and TRI sites may represent modifiable polluting infrastructure that influences older adults’ cognition through distinct pathways. The unexpected finding between highways and visuospatial function among Black older adults may reflect unmeasured effects of highway accessibility (e.g., access to resources, social networks). Future research should explore how older adults interact with their neighborhood to inform interventions that promote cognitive health in older adults.

